# Jasmonate – a blooming decade

**DOI:** 10.1093/jxb/erx068

**Published:** 2017-04-06

**Authors:** Ziqiang Zhu, Richard Napier

**Affiliations:** 1 College of Life Sciences, Nanjing Normal University, Nanjing, China; 2 School of Life Sciences, University of Warwick, Coventry, UK

**Keywords:** Defence responses, development, jasmine (*Jasminum* spp.), jasmonate, JAZ protein, signalling, transcriptional regulation

**The plant hormone jasmonate not only helps plants to defend against necrotrophic fungi and insect attacks, but also regulates many growth and developmental events. The landmark breakthrough in research in this field was the identification of JAZ proteins in 2007, providing the long-sought central signalling molecules bridging perception to downstream transcriptional regulation. Recognizing 10 years of research on JAZ proteins, the reviews in this special issue celebrate what we have learnt, interpret recent discoveries, and provide stimulating new perspectives.**

When walking in the garden, the fragrance of jasmine flowers (*Jasminum* spp.) gives us pleasure (Box 1). The dominant chemical is the methyl ester of (–)-jasmonic acid (JA), an attractant for pollinators but also used by most higher plants as a phytohormone. JA and its derivatives – collectively termed jasmonates – regulate plant seed germination, root elongation, trichome development, anthocyanin accumulation, flowering time, fertility, senescence, and defence responses (Box 2).

**Box 1. *Jasminum sambac***

Jasmine fragrance may be experienced in the garden or in tea. The regulation of fertility by jasmonates includes pollen development, which may provide the link to their use by some flowers as an attractant. Photo reproduced, with permission, from Prof. Huajie Liu.

**Figure:**
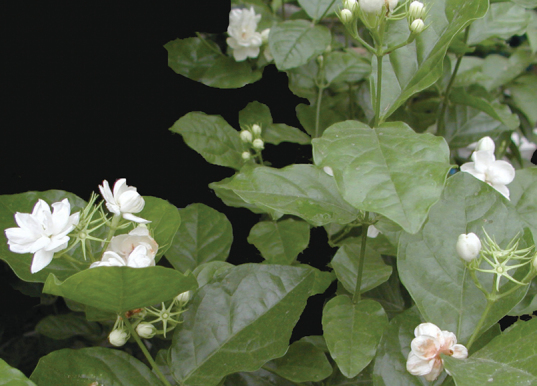

**Box 2. Jasmonates have many roles**

Among their many roles, jasmonates are important in plant defence. This example shows wild-type Arabidopsis (upper panels) compared with JA receptor mutant *coi1-2* (lower panels). The mutant is susceptible to *Botrytis cinerea* infection (left) and almost sterile (right). Images courtesy of Ziqiang Zhu.

**Figure:**
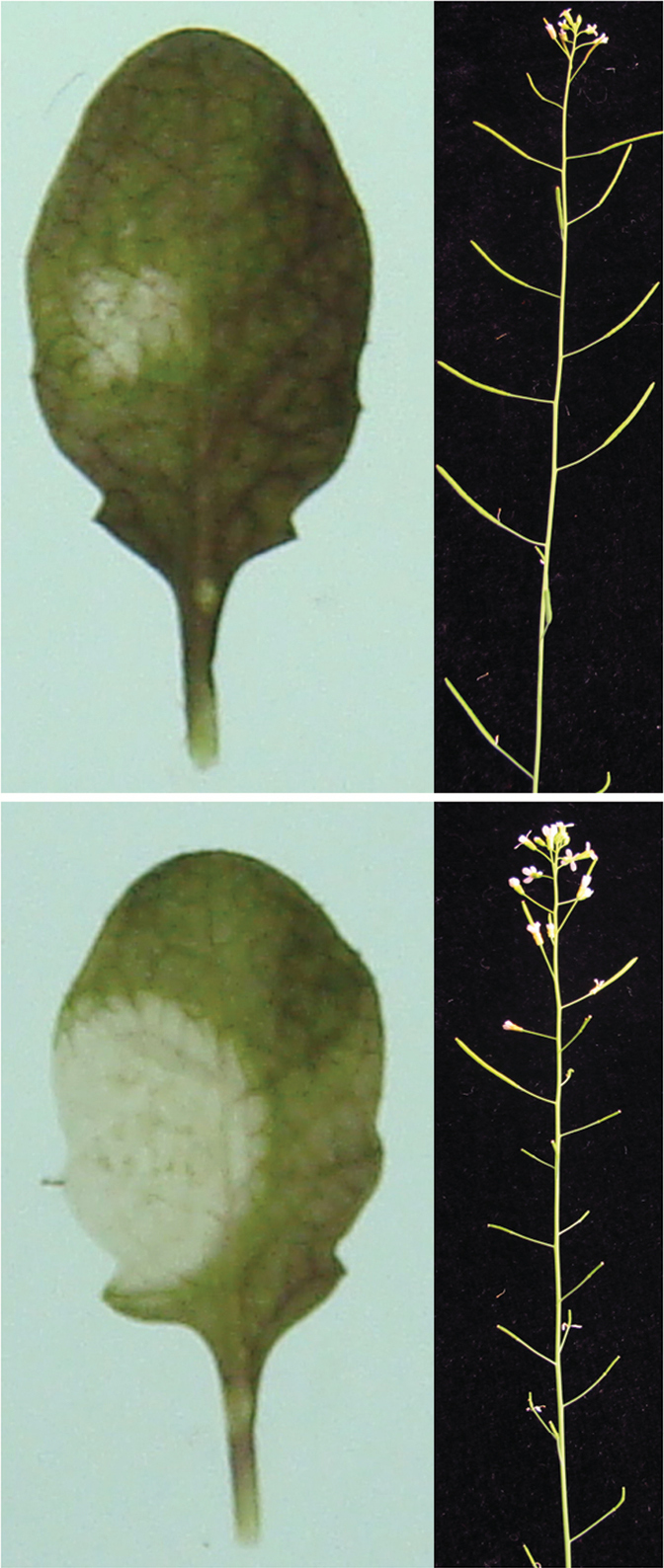

It was only recently revealed that JA is not the ligand that binds the jasmonate receptor: it is converted to its amino acid conjugate (+)-7-iso-jasmonoyl-L-isoleucine (JA-Ile) to become an active signal (Fonseca *et al.*, 2009). Interestingly, a bacterial pathogen (*Pseudomonas syringae*) can produce a mimic of JA-Ile. This mimic, coronatine, strongly activates jasmonate signalling, repressing salicylic acid (SA) accumulation and hijacking the plant’s immune system (Yan and Xie, 2015). Recent advances have identified a number of other pathogens that produce jasmonate analogues as a means to subdue or subvert host defences (Zhang *et al.*, 2017), adding to the network of reactions associated with JA biosynthesis (Wasternack and Song, 2017).

## A flowering of research

Although the past decade has seen much progress following the identification of JAZ proteins, jasmonate biosynthesis and metabolism have been studied for considerably longer. In this issue, Wasternack and Song (2017) bring this extensive field of research up to date and, in parallel, there is lively speculation about the ancestry of biosynthetic pathways, in particular the origins of the conjugation of JA to JA-Ile (Han, 2017). Homologues of all the enzymes of JA-Ile biosynthesis and catabolism are present throughout land plant lineages, suggesting that JA signalling might predate this evolutionary step. However, it may not be by much, since JAR (JA-amino acid synthase) homologues have only been detected in some Charophytes. On the other hand, candidate COI1 (Coronatine Insensitive 1, the jasmonate receptor) sequence homologues appear in more-ancient lineages. Han concludes that JA signalling arose after the colonization of land by plants, but this is clearly an area where more research is needed.

Despite a long history, it is only recently that we have started to discover the detailed mechanisms of JA perception. The discovery of *COI1* was initially reported in 1994, and the mutated gene was cloned in 1998 (Xie *et al.*, 1998). *COI1* encodes a leucine-rich repeat (LRR) type F-box protein that assembles in an SCF protein complex; this targets substrate proteins for degradation, a process which initiates signalling (Dai *et al.*, 2002; Devoto *et al.*, 2002; Xu *et al.*, 2002). Suppressor screens and protein complex purification approaches failed to find the COI1 substrate(s) (Devoto *et al.*, 2002; Xiao *et al.*, 2004), although it was only a few years later that the crystal structure of COI1 in complex with its ligand JA-Ile and its co-receptor (a jasmonate ZIM-domain, JAZ, protein) was solved (Sheard *et al.*, 2010). Some summary images of this beautiful complex are presented by Hu *et al.* (2017), along with a discussion of the latest work illustrating JAZ–MYC interactions and how direct competition between the JAZ repressors and transcriptional co-activators might tighten control over JA-mediated responses.

The basic helix-loop-helix (bHLH) type transcription factor MYC2 was identified as necessary in JA signalling just over a decade ago (Lorenzo *et al.*, 2004). *myc2* mutants are partially insensitive to JA in the commonly used root-growth inhibition bioassay, as well as being susceptible to insect pests. Microarray analysis revealed a genome-wide view of the Arabidopsis transcriptome regulated by MYC2 (Dombrecht *et al.*, 2007). Much recent research on the bHLH family has drawn attention to the family members that elicit specialized metabolic pathways in response to JAs (Goosens *et al.*, 2017). There is immense interest in exploitation of such switchable pathways where they mediate the biosynthesis of high-value chemicals. A number of important pharmaceutical products are plant protectants, induced in response to trauma via JA signalling, making both JA and JA signalling attractive synthetic biology targets. However, historically we needed to learn about JAZ proteins before we could understand how bHLH proteins were linked to COI1.

## Three new blooms – the JAZ proteins emerge

In 2007, three groups independently reported their findings on JAZ proteins. John Browse’s team investigated rapid JA-responsive genes and focused on a protein family with unknown functions. Although loss of function or overexpression of each gene did not alter JA responses, overexpression of a truncated *JAZ1* exhibited JA insensitivity. Further analysis showed that JA triggers JAZ1 degradation and stimulates interaction of COI1 with JAZ1 (Thines *et al.*, 2007). Roberto Solano’s group cloned the mutated gene from an old JA-insensitive mutant (*jai3*) and found that the last two *JAZ3* exons were absent in *jai3*. They further demonstrated that JAZ3 interacts with COI1 and MYC2 (Chini *et al.*, 2007). Edward Farmer’s lab overexpressed a list of JA/wounding-inducible candidate genes and found that *JAZ10* overexpression caused JA insensitivity. Interestingly, they also noticed that *JAZ10* had three different transcript isoforms caused by alternative splicing, but overexpression of the full-length cDNA conferred little difference (Yan *et al.*, 2007).

Biochemical and structural biology studies demonstrated that the COI–JAZ complex is the co-receptor for both JA and coronatine perception (Katsir *et al.*, 2008; Yan *et al.*, 2009; Sheard *et al.*, 2010). Alain Goossens’ group performed immuno-precipitation mass spectrometry to search for new JAZ-interacting proteins and identified one, NOVEL INTERACTOR OF JAZ (NINJA), as a bridge molecule for recruiting TOPLESS to fulfil its transcriptional repression activities (Pauwels *et al.*, 2010). TOPLESS was previously demonstrated to be a transcriptional repressor through the action of histone deacetylation (Long *et al.*, 2006). Additionally, it was recently found that JAZ3 repressed the interaction between MYC3 and the MED25 subunit of the transcriptional co-activator complex known as Mediator, giving another layer of transcriptional repression (Zhang *et al.*, 2015).

Other JAZ-interacting transcription factors serve as hubs to connect jasmonate with other signalling cues, such as ethylene and light (Kazan and Manners, 2011; Hsieh and Okamoto, 2014; Zhu, 2014). However, it is clear that much is still being learnt about the interactions between JA signalling and crosstalk with these cues, as well as with other phytohormones (Huang *et al.*, 2017). Hu *et al.* (2017) also reveal the importance of crosstalk between transcriptional regulators in responses during controlled leaf senescence and cold-stress responses. Very recently, for example, it was shown that JA triggers a transcriptional regulator known as CBF (C-repeat Binding Factor) to up-regulate cold-tolerance genes. Whilst much is being learnt from transcriptomics, it is also encouraging to realize that rational design of novel JA antagonists is starting to provide new tools to help us understand and regulate plant development.

## Jasmonate research buds. What’s next?

There are currently 13 JAZ members in Arabidopsis. However, whether the action of these proteins is completely redundant or not is still to be fully determined. A recent report shows that JAZ2 is specifically expressed in guard cells to control the coronatine-regulated stomatal re-opening process (Gimenez-Ibanez *et al.*, 2017). This study implies that different JAZ members might act in a tissue-specific manner. Tissue- or organ-specific signalling is also indicated by the analysis of *ninja* mutants. It has been reported that NINJA mainly functions in roots to mediate transcriptional repression (Acosta *et al.*, 2013). Thus, further studies on organ- or tissue-specific jasmonate signalling would generate more novel insights. (See also the Insight article by Shyu, 2016, looking at recent studies on the biological roles of individual JAZs through the example of JAZ7: Thatcher *et al.*, 2016; Yu *et al.*, 2016).

One of the most exciting advances in JA signalling has been the exploitation of the unstable nature of JAZ proteins to design a fluorescent molecular sensor that directly monitors JA dynamics *in planta* (Larrieu *et al.*, 2015). This now places JA alongside auxin and abscisic acid in having dynamic, *in vivo* biosensors available, which will undoubtedly promote progress in deconvoluting JA signalling networks and screening for new rationally designed JA analogues (Wasternack and Song, 2017). Yet, binding to COI1 is only one of the transient partnerships made by the JAZ-interacting transcription factors. Indeed, JAZ proteins are implicated in many relationships. For example, MYC2 and ETHYLENE INSENSITIVE3 (EIN3) can physically interact and repress each other, controlling apical hook development and herbivore resistance (Song *et al.*, 2014; Zhang *et al.*, 2014). In addition, MYC2, MYC3 and MYC4 interact with MYB21 and MYB24 to form a cooperative complex to regulate stamen development (Qi *et al.*, 2015). The interrelationships between the JAZ-interacting transcription factors so far identified – more than a dozen proteins – are clearly worthy of further investigation.

Signal crosstalk between JA and other plant hormones or environmental factors is still a developing field. Systematically analyzing protein–protein interactions or protein–DNA affinities will provide important clues for these studies. Additionally, with numerous transcriptomic and/or proteomic data sets, systems biology and mathematical modelling techniques will increasingly be required to integrate such large-scale analyses for understanding the signalling network.

Epigenetic regulators have also been suggested to be involved in JA signalling. For example, plants impaired in HISTONE DEACETYLASE6 (HDA6) exhibit altered expression of JA-responsive genes (Wu *et al.*, 2008). Moreover, HDA6 was detected in the COI1 protein complex via immunoprecipitation (Devoto *et al.*, 2002). These reports suggest that histone deacetylation is required for the JA response, and it will be exciting to find out how successfully a plant can pass on tolerance against pest, pathogen or cold stress to future generations, or how much additional genetic variability in tolerances to these stresses may be unmasked as we learn to manage epigenetics.

Last but not least, how to manipulate plant fitness and improve yields with the knowledge we have gained? A point mutation (A384V) has already been introduced into COI1 protein to reduce its affinity with coronatine but not affect its binding with JA for enhancing plant resistance to bacterial infection (Zhang *et al.*, 2015*b*). Combining similar strategies with CRISPR-Cas technology will shed new light on future precision breeding (Kumar and Jain, 2015).
